# Book Reviews

**Published:** 1903-12

**Authors:** 


### BOOK REVIEWS.

I. The Practical Medicine Series of Yearbooks, comprising ten volumes in the year's progress in medicine and surgery. Issued monthly. Under the general editorial charge of Gustavus P. Head, M. D., Professor of Laryngology and Rhinology in the Chicago Post-Graduate Medical School. Vol. VI. General Medicine. Edited by Frank Billings, M. S., M. D., head of the Medical Department and Dean of the Faculty of Rush Medical College, Chicago. And J. H. Salisbury, M. D., Professor of Medicine, Chicago Clinical School. Duodecimo, pp. 316. Chicago: The Year Book Publishers. 1903. (Price, $1.50; entire series, $7.50.)

II. Volume VII. Pediatrics. Edited by Isaac A. Abt, M. D., Assistant Professor of Medicine, Rush Medical College. Orthopedic Surgery. Edited by John Ridlon, M. D., Professor of Orthopedic Surgery, Northwestern University Medical School. Chicago: The Year Book Publishers. 1903. (Price, $1.25; entire series, $7.50.)

I. This book gives a most satisfactory résumé of the advances in general medicine during the past year (1902), and presents it in a form both attractive and available for the busy physician. Diseases of the gastrointestinal tract necessarily take up considerable space, which is well appropriated. Reference is made to Deaver's exhaustive article on pancreatic disease, published in the *Am. Jour. Med. Sci.*, February, 1903; also to Opie's work in this field.

II. The portion of Volume VII., devoted to the consideration of pediatrics is full of interest for every physician who is responsible in any manner for the care of infants. It contains a reference to J. W. Ballantyne's article in the *British Medical Journal* on the premature infant, and to Irving M. Snow's case of tetany complicating diphtheria, published in the *Am. Jour. Med. Sciences,* December, 1902. It is replete with valuable material, relating to diseases of the digestive organs and of nutrition,—two of the most important topics pertaining to infantile disease.

In the section on orthopedic surgery Ridlon gives the Lorenz method of dealing with congenital dislocation of the hip, and with club foot, and makes detailed reference to the visit of Professor Lorenz to this country in 1902, and to the work he performed. Much of the other material will be found of timely interest to all who practise orthopedic surgery.

These two volumes are excellent examples of what can be done by men trained in the art of condensing valuable medical material and serving it up in its best form.

---

A Textbook of the Diseases of Women. By Thomas A. Ashby, M. D., Professor of Diseases of Women in the University of Maryland. Octavo, 661 pages, with 233 illustrations. Baltimore: Williams & Wilkins Co. 1903.

Notwithstanding the pronunciamento of Howard Kelly that gynecology is becoming, or will become, a lost art, all of its value having been absorbed by the general, all around, sure enough surgeons, more new textbooks and treatises on diseases of women have issued from the press this season than in many previous autumns. Publishers do not print books for their own amusement, and if they turn them out at all it is because they are wanted. After squeezing a lemon and using all its delicious succulence, it is not a generous act to girdle the tree that bore the fruit, and we predict that the attempt to strangle gynecology by those who have profited most in its practice will react upon them to their dismay. We are prompted to preface our comments on Professor Ashby's excellent book with the foregoing remarks, to point out the wisdom of the homely, but sensible, proverb "always speak well of a bridge that carries you safely over the stream."

The time has passed, at least nearly so, when a critical review of a work is needed. What is most required is a statement as to the practical value of a book based upon its careful examination by a competent observer. This one begins with a historical sketch of gynecology, of which we quite approve. There is too much neglect of the history and traditions of medicine nowadays, and we hope to see this corrected through the efforts of such competent men as Dr. Ashby. Every textbook, every teacher, no matter as to the topic, should impart to the student some information, even though it be meager, of the history of the subject written upon or taught.

The author of this work gives due prominence to physical diagnosis, to which end he describes in detail the modern methods of examination. The various postures are portrayed by words and illustration, and necessary instructions are given for a thorough exploration of the pelvis and its contents. The chapter on gynecological technic, embracing surgical asepsis, antisepsis, and sterilisation, is a well digested presentation of a most important topic. All the various diseases of the female genital tract, from vulva to ovary, are set forth in concise, practical, yet sufficiently complete form, and are made clear to the student, which is tantamount to saying that everybody who reads may learn what the author is teaching.

Operative gynecology, including plastic surgery of the perineum and cervix and of fistulæ, neoplasms of the uterus, diseases of the tubes and ovaries, together with affections of the urethra, bladder and ureters, are all adequately presented and the word-pictures are in many instances most charming. There is room, however, in this part of the work, especially where it deals with diseases of the ovary and of the urinary tract, for more complete art illustration. Ashby's description and treatment of cystitis, acute and chronic, will prove of special aid and value to the young practitioner, who usually finds these conditions troublesome to manage, and will welcome such clear and concise directions as he will find in this book.

The size and make-up of this work is attractive, it being convenient to handle and easy of reference. We doubt not, it will receive favor at the hands of students and junior physicians, as well as trained gynecologists.

TRANSACTIONS OF THE MEDICAL SOCIETY OF THE STATE OF NEW YORK. Ninety-seventh Annual Meeting, held at Albany, N. Y., January 27, 28 and 29, 1903. Frederic C. Curtis, M. D., Secretary. Brandon Printing Co., Albany. 1903.

This volume is an interesting record of the proceedings of this society at its ninety-seventh annual meeting. Few societies can show a continuous existence for nearly a century, and none has published so complete an account of its work during all this time. The advantages of an annual volume of society transactions are so superior to the journal method, it is surprising that it is not adhered to, at least by the older societies. Journals become lost, mislaid, or otherwise disposed of, and thus the record is broken; whereas, a book properly bound is always accessible, and is an ornament to the library. It is the cheaper method, too; hence it does not necessitate large fees. The journal method will bankrupt any society in the end, unless it has a large constituency paying in turn large dues to support it. There are too many journals in existence already, and state societies should not enter the field of competition, because it places them too distinctly in the commercial field and serves to lower their *esprit de corps*. In the present volume the president's address entitled, Progress, liberty, unity, is a state paper of more than ordinary importance, and one that reflects the highest honor upon its distinguished and scholarly author, Dr. Henry Reed Hopkins, of Buffalo. The address by Professor Charles A. L. Reed, of Cincinnati, entitled, Rudolf Virchow; an appreciation, is a world-famous paper that has attracted attention wherever the name and fame of Virchow are known.

The scientific contributions are above the average of professional papers. The discussions are rather meager,—a defect that is easily remedied. Fewer papers would mean a less crowded program, and more opportunity for discussion. The society should remain in session three full days, which would tend to relieve the pressure on the program. We miss the list of delegates and permanent members in attendance, an omission that should be remedied, because it is important to know who were present at each meeting. The editorial work of the book reflects the highest credit upon the secretary.

---

American Textbook of Surgery. For Practitioners and Students. Edited by William W. Keen, M. D., Professor of the Principles of Surgery and of Clinical Surgery, Jefferson Medical College, Philadelphia; and J. William White, M. D., Professor of Surgery, University of Pennsylvania, Philadelphia. Fourth edition, thoroughly revised and greatly enlarged. Octavo, 1363 pages, with 551 text-illustrations and 39 full-page plates, many in colors. Philadelphia, New York, London: W. B. Saunders & Company. 1903. (Cloth, $7.00 net; sheep or half morocco, $8.00 net.)

The reissue of this standard work will afford an opportunity for the medical profession to obtain one of the most thoroughly useful works on surgery that has been published in this country. The remarkable sale of the previous editions has been a subject of comment, and must have been gratifying to editors, contributors, and publishers.

This fourth edition has been carefully revised to keep it abreast of the work of American surgeons. The editors gracefully state that since the last edition was issued Abbe, Crile, Coley, Cushing, (of Baltimore), Da Costa, Edebohls, Finney, Fowler, Frank, Frazier, Halsted, Mayo, Matas, Monks, Stewart, Wyeth, Wier and many others have made important contributions to surgery in this country, while many European surgeons have done the same, and that all the contributions have been utilised in this book. This surgical activity has possibly been stimulated in a considerable degree by this work,—a modest claim the authors, in our view, are justified in making.

Among the many improvements in this edition are six new chapters, the subjects of which are military surgery, naval surgery, tropical surgery, examination of the blood, immunity and the pancreas. The latter receives more extended notice in order to cover the surgery of that organ, which has taken its place in surgical literature since the publication of the last edition. The work presents the same general appearance as formerly, and will maintain its place in literature as the chief exponent of American surgery.

---

THE PRINCIPLES OF OBSTETRICS. A Practical Manual for the Student and General Practitioner. By STANLEY PERKINS WARREN, M. D., Portland, Me., Obstetric Surgeon to the Maine General Hospital. Octavo; 385 pages. Illustrated by 165 lines and half-tone engravings. William Wood & Company, New York. 1903. (Cloth, $3.00; leather, $3.75 net.)

There is no drought in obstetric literature this season and, it must be confessed, much of it is of the very best. This book, for example, though not as pretentious as some others is, nevertheless, one of the best of its class. It does not claim place among the exhaustive treatises, nor does it belong with the smaller manuals, but it occupies an intermediate position, being a book that contains the essentials of obstetrics without being voluminous on the one hand, nor elementary on the other. Moreover, its price places it in a class practically by itself, and meets the conditions often confronted in which a limited purse must obtain much for its outlay.

The author has exhibited discretion in selecting his material and in arranging it for the practical obstetrician. It is a book that can be consulted quickly in an emergency, and in which will be found just what is required,—namely, guidance in a dilemma when aid is most needed. Considerable attention is given to obstetrical asepsis in private practice, which is a topic of great importance. The book is adequately illustrated with well executed drawings and engravings, and is a commendable addition to the literature of obstetrics.

---

STUDIES IN THE PSYCHOLOGY OF SEX. SEXUAL IMPULSE IN WOMEN. Third Volume in Series. By HAVELOCK ELLIS, L. S. A. (England), General Editor of the Contemporary Science series since 1899. Sold only to physicians, lawyers, clergymen, advanced teachers, and scientists. Philadelphia. F. A. Davis Company. 1903. (Cloth, $2.00 net, delivered.)

The subject dealt with in this book is one of great delicacy and deserves the most patient study. In its presentation, too, there should be equal delicacy of expression, with an elimination of everything that should excite prurience or create disgust. Some portions of this book are constructed on scientific lines and will serve to throw light on a darkened field,—one at all events too little understood and, perhaps, greatly misunderstood.

Some other parts of the book might have been omitted without, it seems to us, detracting from its usefulness or scientific value. We refer to some of the "histories" that are given with such graphic exactitude of detail, as to reach pretty near the borderline of decency. The attempt to limit the sale of this series of "studies" is wise, though it is doubtful if it succeeds. The announcement of the fact that only physicians, lawyers, clergymen, advanced teachers, and scientists may become purchasers is quite sufficient to sharpen the appetite of the curious who, no doubt, will buy surreptitiously in large numbers.

---

A Thesaurus of Medical Words and Phrases. By Wilfred M. Barton, M. D., Assistant to Professor of Materia Medica and Therapeutics, and Lecturer on Pharmacy, Georgetown University, Washington, D. C.; and Walter A. Wells, M. D., Demonstrator of Laryngology and Rhinology, Georgetown University, Washington, D. C. Octavo, 534 pages. Philadelphia, New York, London: W. B. Saunders & Company. 1903. (Flexible leather, $2.50 net; with thumb index, $3.00 net.)

This is a medical wordbook constructed on new lines as concerns the field of medicine, though similar works cover general literature, for example Roget's, which is so useful. The medical writer or speaker will find this "thesaurus" a great aid in increasing his vocabulary or simplifying his expressions. It is most important for a speaker or writer to convey to his audience the exact thought of his brain in words so plain that he may not be misunderstood. Very often the real point of an argument or theme is missed through failure to choose the appropriate phrase. This book will serve to correct such faulty diction, if referred to frequently and with care, whenever there is doubt. It is superbly printed and handsomely bound in flexible red leather.

---

The Latin Grammar of Pharmacy and Medicine. By D. H. Robinson, Ph. D., late Dean of School of Arts, and Professor of Latin Language and Literature, University of Kansas. With an introduction by L. E. Sayre, Ph. M., Professor of Pharmacy in, and Dean of, Department of Pharmacy, University of Kansas. Fourth edition. Thoroughly revised by Hannah Oliver, A. M. Philadelphia: P. Blakiston's Son & Company. 1903. (Price, $1.50.)

In order to acquire sufficient knowledge of Latin to read or write it correctly, it is necessary to take up its study deliberately and pursue it systematically under a competent tutor. There is no royal road to learning; it must be obtained through patient and enduring effort. The book before us evidently has been prepared by a student of language and a scholar of no mean attainments, and as a textbook for the beginner of medical Latin, it is unique and superb. As a work of reference for older persons who may have become "rusty" in the classics it also is of great value. Therefore, it is a book that may find an appropriate place in the outfit of both student and physician.

---

THE PRACTICAL MEDICINE SERIES OF YEAR BOOKS. Ten volumes. Issued under the general editorial charge of GUSTAVUS P. HEAD, M. D., Professor of Laryngology and Rhinology in the Chicago Post-Graduate Medical School. Volume IX. Physiology, Pathology, Bacteriology, Anatomy, Dictionary. Edited by W. A. Evans, M. D., Adolph Gehrmann, M. D., William Healy, M. D. Duodecimo, pp. 233. Chicago: The Year Book Publishers. 1903. (Price, $1.25; entire series, $7.50.)

This might well be termed a conglomerate number of this useful series. It deals with a number of different topics, yet all are more or less related. In it will be found the recent advances pertaining to physiology, pathology, bacteriology, and anatomy, while to it is added a dictionary of new medical terms. This latter point is an excellent idea, novel in plan, and practical in execution. New terms are constantly appearing in print that are not found in the standard dictionaries, hence this method of reinforcing the wordbook outfit is commendable. It is in keeping with the progressive spirit of the editors.

---

A MANUAL OF OBSTETRICS. By A. F. A. KING, M. D., Professor of Obstetrics and Diseases of Women, in the Medical Department of the Columbian University, Washington, D. C., and in the Medical Department of the University of Vermont. Ninth edition, revised and enlarged. In one 12mo volume of 628 pages, with 275 illustrations. Lea Brothers & Co., Philadelphia and New York. 1903. (Cloth, $2.50 net.)

This manual is a familiar friend, having appeared at intervals for more than 20 years. It is a rare circumstance for a work on obstetrics to pass to its ninth edition during the active lifetime of its author, yet such has been the case with this manual, and Professor King himself has prepared each edition for the press. He has kept his excellent book abreast of the progressive present, and its popularity is unabated. Indeed, it is one of the best, as it is one of the oldest obstetric manuals, and is not only a superb guide for the student, but it is a valuable reference book for the obstetrician.

---

RADIUM AND OTHER RADIOACTIVE SUBSTANCES. With a Consideration of the Treatment of Disease by the Ultraviolet Light. By WILLIAM J. HAMMER, Consulting Electrical Engineer. Duodecimo, pp. 72. Illustrated. New York: D. Van Nostrand Co. 1903. (Price, $1.00.)

This monograph, being in reality a lecture delivered by the author before a joint meeting of the American Institute of Electrical Engineers and the American Electrochemical Society, at New York, April 17, 1903, is full of interest for all who are engaged in the study of radium and other radioactive substances.

It also presents a consideration of phosphorescent and fluorescent substances, together with the properties of selenium, and closes with a short section on the treatment of disease by the ultraviolet light. Mr. Hammer has condensed into small space much valuable material.

PROGRESSIVE MEDICINE. Fifth Annual Series. Volume III. September, 1903. A Quarterly Digest of Advances, Discoveries and Improvements in the Medical and Surgical Sciences. Edited by HOBART AMORY HARE, M. D., Professor of Therapeutics and Materia Medica in the Jefferson Medical College of Philadelphia. Octavo, 398 pages; illustrated. Philadelphia and New York: Lea Brothers & Co. (Per volume, $2.50, by express prepaid. Per annum, in four cloth-bound volumes, $10.00.)

The prosperity of this digest of progress in medical science is dependent upon its excellence, it being the only quarterly of its kind, and it fills the place for which it was designed. In this volume may be found diseases of the thorax and its viscera, including the heart, lungs, and bloodvessels, by William Ewart; dermatology and syphilis, by William S. Gottheil; diseases of the nervous system, by William G. Spiller; and obstetrics, by Richard C. Norris. It is doubtful if so much valuable material has ever before been put into such compact form, and sold at such a low price.

---

THE MEDICAL NEWS VISITING LIST FOR 1904. Lea Brothers & Company, Philadelphia, 706 Samson Street; New York, 111 Fifth Avenue.

This is the eighteenth annual issue of one of the most useful articles in a physician's equipment. To do without a visiting list would be next to impossible in these hurrying days. The loss in income from unrecorded visits and other omitted memoranda, did one not have such a book in the pocket, would be surprising if accurately computed. This list has been revised and contains all that is needed for business purposes, besides much important clinical material for quick reference. It is issued in four styles in order to meet the various requirements of physicians. The weekly, monthly and 30 patient perpetual, contain 32 pages of data and 160 pages of classified blanks. The 60-patient perpetual consists of 256 pages of blanks alone. Each constitutes one wallet-shaped book, bound in flexible leather, with flap and pocket, pencil and rubber, and calendar for two years, $1.25. Thumb-letter index, 25 cents extra. By mail, postpaid to any address.

---

A Laboratory Guide in Urinalysis and Toxicology. By R. A. Witthaus, M. D., Professor of Chemistry, Physics and Toxicology in the Medical Department, Cornell University, New York. Fifth edition. New York: William Wood & Co. 1903. (Price, $1.00.)

Laboratory work is an essential part of a student's preparation for his life occupation, and it should be done with serious application, under a competent instructor. Not only this, but laboratory guides are an essential part of the equipment. This one is on a practical subject prepared by a practical teacher, has stood the test of time, and will be found of vast usefulness, not only to the student, but to the practitioner. It is so important to be prepared to make an elaborate analysis of urine for clinical purposes, that no argument is needed to prove the fact; in other words, this book speaks for itself.

---

Lea's Series of Medical Epitomes. Medical Jurisprudence. A manual for Students and Practitioners. By E. W. Dwight, M. D., Instructor in Legal Medicine, Harvard University. In one 12mo volume of 249 pages. Series edited by V. C. Pedersen, A. M., M. D., Instructor in Surgery at the New York Polyclinic Medical School and Hospital. Philadelphia and New York: Lea Brothers & Co. 1903. (Cloth, $1.00, net.)

The author of this compend, or epitome as it is called, has accomplished a difficult task in presenting the essentials of medical jurisprudence in a small compass, and yet preserving the important features for use in the class room. Only such an experienced teacher as Dr. Dwight could be expected to attain such results. The book deals with a subject that is too often neglected, and this form of presentation ought to do much toward stimulating a familiarity with it.

---

Medical Directory of New York, New Jersey and Connecticut. Published by the New York State Medical Association. Volume V. 1903.

This volume appears promptly and maintains the high standard established in the publication of this directory, as well as the same general plan of the preceding volumes. Tinted paper in some of its sections has been found so practical that it is continued. An addition has been made by publishing a list of medical libraries, which may prove useful. The total number of names in the book is 14,524, of which 11,350 belong to New York; 1,991 to New Jersey, and 1,183 to Connecticut. Of those in the first group 5,589 reside in Greater New York, and 5,761 in the state at large outside the greater city.

---

The Practical Medicine Series of Year Books. Ten volumes. Issued under the general editorial charge of Gustavus P. Head, M. D., Professor of Laryngology and Rhinology in the Chicago Post-Graduate Medical School. Volume VIII. Materia Medica and Therapeutics; Preventive Medicine; Climatology; Suggestive Therapeutics; Forensic Medicine. Edited by Drs. George F. Butler, Henry B. Favill, Norman Bridge, Daniel R. Brower, and Harold N. Moyer. Duodecimo, 326 pages. Chicago: The Year Book Publishers. 1903. (Price, $1.50; entire series, $7.50.)

The topics considered in this volume are such as to appeal to the general practitioner of medicine, and they are prepared by authors of experience in the several fields that are therein cultivated. There is nothing new to be said regarding the publication of this series of yearbook literature. It has established itself as a useful aid to the busy doctor, and it is maintaining a standard of excellence that will continue to attract professional interest.

Blakiston's Quiz Compends. A Compend of Human Anatomy. By Samuel O. L. Potter, M. D., Author of the Handbook of Materia Medica, Pharmacy and Therapeutics. Seventh edition, revised and enlarged. Illustrated. Philadelphia: P. Blakiston's Son & Co. 1903. (Price, 80 cents.)

The study of anatomy involves patient effort and draws heavily upon the memory. Any well-matured scheme that will aid the student in memorising the essentials of the topic is to be commended. Anatomy is especially adapted to presentation in the compend form, and we heartily recommend this one to students who are pursuing anatomical studies.

THE PHYSICIANS' POCKET ACCOUNT BOOK. By J. J. TAYLOR, M. D. The Medical Council, Philadelphia. (Price, $1.00.)

This is a new edition, reduced in size, of the pocket account book published by Dr. Taylor for two years past. For some reasons the size of this edition may prove an advantage, chiefly because it can be carried in the pocket with greater convenience. This system of bookkeeping is a good one and it could be adopted by most physicians with advantage.

